# Medical, subjective and objective forms of exercise dependence and the role of learning, cognitive and emotional biases

**DOI:** 10.1177/13591053241304561

**Published:** 2024-12-23

**Authors:** Kate Nicholls, Philip Dean, Jane Ogden

**Affiliations:** University of Surrey, UK

**Keywords:** consequences, exercise, exercise addiction, exercise dependence, punishment sensitivity, reward sensitivity

## Abstract

Despite numerous benefits of regular exercise, research has demonstrated some people develop problematic exercise behaviour, with ongoing debates regarding the definition. This study defined three approaches: a traditional medical model including for example withdrawal symptoms; a subjective approach whereby individuals identify their own problematic exercise; and an objective perspective involving persistent exercise despite negative consequences. This cross-sectional study assessed the association between these three approaches in UK-based frequent exercisers (*n* = 139) alongside correlations with learning, cognitive and emotional biases (reward vs punishment sensitivity, delay discounting and sensation seeking). The results indicate these three approaches to problematic exercise are related but different. Further, medical problematic exercise was associated with heightened sensitivity to reward and punishment; subjective problematic exercise was only associated with heightened punishment sensitivity; objective problematic exercise was associated with reduced punishment sensitivity. This novel classification approach to problematic exercise may help clarify the factors that initiate and perpetuate this behaviour.

## Introduction

Regular exercise has been shown to provide many benefits, including, but not limited to, the reduced risk of major illnesses, improved mood and better quality of sleep (Banno et al., 2018; Lear et al., 2017; Liao et al., 2015; Moore et al., 2016). In line with this, national guidelines state that adults should aim for a minimum of 150 minutes of moderate-intensity exercise per week (NHS, 2021). For a minority of people, however, a more problematic relationship with exercise can develop, whereby the person continues to exercise at the expense of other areas of life. Research has labelled problematic exercise using a number of terms including compulsive exercise, exercise addiction, excessive exercise and problematic exercise and has been measured using a number of different assessment tools such as the Exercise Addiction Inventory (EAI; Griffiths, 2005), the Compulsive Exercise Test (CET; Taranis et al., 2011), the Exercise Dependence Scale (EDS; Hausenblas and Downs, 2002) and the Exercise Dependence Questionnaire (EDQ; Ogden et al., 1997), (see Cook et al., 2014 for a comprehensive review). Due to the array of terminology, for the purpose of the present study the term ‘problematic exercise’ will be used. Further, research has also explored the relationship between problematic exercise and eating disorders and illustrates a clear overlap with problematic exercise being both primary and secondary to eating related problems (Cook et al., 2014; Coverley Veale, 1987; Cunningham et al., 2016). When problematic exercise co-occurs with disordered eating, it has been found to result in significantly higher levels of physical and psychological distress (Young et al., 2018), and prior research has debated both the existence of primary exercise dependence as a potential disorder (Bamber et al., 2000), and also the potential differing nature of problematic exercise with and without the presence of disordered eating (Adkins and Keel, 2005).

Many exploring problematic exercise have used measurement tools and theoretical perspectives based on the medical model of substance abuse (Freimuth et al., 2011) focusing on withdrawal symptoms, tolerance, increased salience, euphoria, mood modification and excessive time exercising to the extent that it interferes with other responsibilities. Some research, however, highlights alternatives to this medical model approach, such as the role of a subjective perspective emphasising an individual’s own view of their behaviour. For example, whilst Ogden et al. (1997) measured exercise dependence in line with a more medical perspective, they also assessed the individual’s insight into their behaviour which included a subjective awareness of a compulsion to exercise (Coverley Veale, 1987). Likewise, awareness of problematic behaviour is seen as a key feature of the Stages of Change model in addiction treatment programmes (Prochaska et al., 1992) and Moreau et al. (2023) reported that whilst their participants associated with having exercise addiction many did not see their exercise as problematic. Accordingly, a subjective perspective with a focus on self-identified problematic exercise reflects an alternative approach to the medical model and could differentiate between committed exercisers who feel that exercise gives a net benefit to their life, and those who feel that their relationship with exercise is problematic and causes harm.

In contrast, an additional approach would be to take a more objective perspective to problematic exercise. Continuing to exercise in the face of aversive consequences can be interpreted as problematic by others even if the exercisers themselves do not recognise a problem. In line with the definitions used in quality-of-life research, this can be conceptualised as a measure of objective problematic exercise (Bowling, 2001; Ogden, 2023). Prior research into continuation despite the consequences has predominantly come from case study research. One such case study of a 25-year-old martial artist reported problems from over exercise such as getting behind in her coursework and leaving a final university exam early to go and train, the breakdown of her relationships with her partner and friends, lack of concentration, financial debt due to multiple gym memberships and competition fees, extreme anxiety when unable to exercise and repeated injuries (Griffiths, 1997). Another case study of a 42-year old male school teacher reported extreme weight loss, relationship difficulties, feelings of depression, loss of libido and disturbed sleep following persistent exercise (Lyons and Cromey, 1989). Despite these seemingly negative consequences the martial artist was not upset by her relationship break up, and did not associate with being an addict, and the 42-year-old was pleased with his weight loss and felt that his actions were reasonable. Persistent exercise even in the presence of harm is therefore an alternative way to objectively conceptualise problematic exercise drawing upon the views of others. The notion of persistent exercise regardless of consequences is also embedded within some measures of problematic exercise. For example, the CET (Taranis et al., 2011) contains the item ‘I usually continue to exercise despite injury or illness, unless I am very ill or too injured’. Similarly, the EDS (Hausenblas and Downs, 2002), contains items such as ‘I exercise despite recurring physical problems’. These individual scale items typically focus on the physical injuries that can occur from problematic exercise, however, they do not cover the full range of possible consequences which have been highlighted in prior case study research described above.

Problematic exercise can therefore be conceptualised within the medical model, or as either subjective or objective. Research within this field has also highlighted biases which may relate to exercise and whether such behaviour becomes problematic. The second part of this study therefore aimed to assess the link between these three forms of problematic exercise and a learning bias (learning more effectively from reward than from punishment), a cognitive bias (delay discounting), and emotional bias (sensation seeking). Such biases highlight potential mechanisms as to why healthy levels of exercise can become problematic for some people. Each of these biases is discussed below in relation to exercise and problematic exercise.

Whilst the learning bias of sensitivity to reward over sensitivity to punishment has been frequently explored in the context of addiction (Emery and Simons, 2017; Franken and Muris, 2006; Wardell et al., 2015), it has been applied less frequently to exercise. A meta-analysis, however, found that lower sensitivity to punishment is more likely to be found in practitioners of extreme or adrenalin sports, such as sky-diving (McEwan et al., 2019). Likewise, an Australian study compared sensitivity to reward and punishment in severe and non-binge eaters who also showed high levels of problematic exercise (Lyvers et al., 2023). The study found that, similar to the wider addiction literature, higher sensitivity to reward correlated with higher problematic exercise. For participants classified as severe binge eaters, problematic exercise was also associated with lower sensitivity to punishment.

The cognitive bias of delay discounting is common among many substance and behavioural addictions (Kirby et al., 1999; Ledgerwood et al., 2009; Petry, 2001). Delay discounting is the reduction in the perceived value of a reward related to the time taken for the reward to be given. High rates of delay discounting mean participants prefer to forgo a larger reward later for a smaller reward immediately available. Weinsztok et al. (2021) called for further research into the role of delay discounting in proposed behavioural addictions, particularly exercise. Regarding exercise, Daugherty and Brase (2010) used both the traditional monetary choice questionnaire and time perspective measurements of delay discounting (where participants contemplate the immediate and future consequences of a behaviour). They found that exercise is not significantly predicted by the traditional monetary choice delay-discounting measure but instead is positively predicted by both future and present hedonistic perspectives (high focus on the future, and a high focus on in the moment pleasure respectively). Therefore, this current study used time perspective as a measure of cognitive bias for immediate rewards over delayed future rewards.

The third bias relates to emotions, and addiction research also suggests the presence of an emotional bias in sensation seeking. For instance, emotion dysregulation and associated sensation seeking have been linked to the increased negative consequences of alcohol use (Chavarria et al., 2021). In the context of exercise addiction, sensation seeking has been studied in relation to mountaineering, finding that those who are ‘addicted’ to mountaineering report higher levels of sensation seeking, emotion regulation difficulties and higher risk-taking (Habelt et al., 2023). Another study found that paragliders and opioid-dependent participants had equivalent levels of sensation seeking, which were significantly higher than the control group (Franques, 2003). These prior studies suggest that at least for riskier physical activities, there is a similar drive for intense emotional sensations, which could be considered similar to that experienced by people with substance-based addictions.

Research therefore highlights that whilst exercise has many health benefits, excessive exercise can become problematic and may do harm. Research also indicates some links with learning, cognitive and emotional biases. There remain debates, however, as to how problematic exercise should be conceptualised and measured. Further, how different forms of problematic exercise relate to different biases remains unclear. The present study therefore conceptualised problematic exercise in terms of a medical model and subjective or objective problematic exercise and due to the absence of a measure of objective problematic exercise first aimed to develop an appropriate measurement tool. The study next aimed to assess the relationship between these three different approaches to problematic exercise to see if they are inter-related or discrete constructs. Finally, the study aimed to assess the association between the three forms of problematic exercise and the different forms of cognitive bias.

It was hypothesised that the three conceptualisations of problematic exercise would correlate with each other, manifesting as related, but separate measures of problematic exercise. No specific hypotheses were made about the links between types of problematic exercise and specific biases.

## Method

### Design

This study consisted of a cross-sectional online self-report survey. Participants completed a range of surveys, including demographics, details of their exercise routines, measures for the three types of problematic exercise (medical model, subjective and objective) and measures for the three types of bias (reward and punishment sensitivity, present vs future orientation and sensation seeking).

### Participants

Participants who frequently exercised for more than 3 hours per week, lived in the UK, and who were aged 18 and over were recruited online through social media, face-to-face in gyms or in other sports settings. A total of 145 participants completed the survey; however, some participants input impossible (e.g. 111 days per week) or implausible (e.g. 16 hours per day, 7 days per week) amounts of exercise per week; these records were removed (total weekly exercise *z*-score >3), leaving a total of 139 participants with a mean age of 39.68 (see Table 1 for demographic details). All participants provided informed consent and were debriefed at the end of the survey and received no reimbursement or incentive for completing the survey. This study was approved by the university ethics committee.

**Table 1.** Participant demographics.

Gender	Female	69
	Male	70
Ethnic background	Asian or Asian British	4
	Black or Black British	2
	Mixed Ethnic Background	7
	White	123
	Other Ethnic Group	3
Education level	GCSE or equivalent	11
	A Levels or equivalent	13
	Bachelor’s degree or equivalent	42
	Master’s degree or equivalent	44
	Doctorate	19
	Other	10
Employment status	Employed full time	88
	Employed part time	10
	Self employed	18
	Student	13
	Unemployed (looking for work)	3
	Retired	5
	Other	2
Previously sought help	Addiction related problem	1
	Mental health problem	39
	Eating related problem	5
	Mental health and eating related problems	4
	Addiction and mental health problems	1
	Not sought help	89

The most popular sport or type of exercise conducted by these participants was running (*n* = 83), followed by cycling (*n* = 61), weightlifting/weight training (*n* = 51), High Intensity Interval Training (HIIT; *n* = 37), swimming (*n* = 30) and yoga (*n* = 30). Several other physical activities were also included, including team sports, combat sports, dance and aerobic activities. Most participants listed a combination of physical activities with a mean number of 3.14.

## Measures

### Demographics

Participants provided their age, identified gender, ethnic background, highest level of educational attainment and employment status. Participants were also asked whether they had ever previously sought help for addiction, mental health or eating-related problems. They were then asked to confirm their regular physical activities from a list of 40 exercise types, including running, cycling, weightlifting, hiking, team sports, combat sports and typical exercise classes, such as aerobics or spinning.

### Weekly exercise

The short-format, self-administered version of the International Physical Activity Questionnaire (IPAQ; Craig et al., 2003) was used to assess the duration and intensity of typical weekly exercise. As the focus of the study was on intentional exercise, the ‘time spent sitting domain’ was not included. For both vigorous and moderate activity, days per week were multiplied by hours per day to create weekly vigorous exercise and weekly moderate exercise totals. The vigorous and moderate total weekly hours were then added to create the total weekly hours.

### Types of problematic exercise

#### Medical model of problematic exercise

To assess a medical model of problematic exercise this study utilised two subscales from the Exercise Dependence Questionnaire (EDQ; Ogden et al., 1997): Withdrawal and Interference. These subscales include items such as ‘If I cannot exercise I feel I cannot cope with life’, and ‘the rest of my life has to fit in around my exercise’. These subscales were interpreted as the closest to the standard medical model-based addiction criteria, and in this study, they had adequate internal reliability (Cronbach’s alpha = 0.77).

#### Subjective problematic exercise

To assess subjective problematic exercise this study used the insight subscale of the EDQ (Ogden et al., 1997) which includes statements such as ‘my exercise is ruining my life’, in combination with the standalone question ‘Do you feel that your level of exercise is a problem?’, for which participants answered on a 5-point scale from definitely not to definitely yes. This combined scale also has adequate internal reliability (Cronbach’s alpha = 0.70).

#### Objective problematic exercise

Some existing measures of problematic exercise include items which relate to objective exercise and the continuation of exercise in the face of negative consequences. For example, the Obligatory Exercise Questionnaire (Pasman and Thompson, 1988), has the item ‘I have exercised when advised against such activity (i.e. by a doctor, friend)’. However, for the purpose of the present study these single items were deemed too narrow as they don’t fully capture the breadth of ways in which negative consequences can both occur and be ignored. As a result, for the purposes of the present study, a new scale was developed to capture exercise persistence despite the negative consequences which can arise from a problematic relationship with exercise. Initially, we identified and explored existing measures including the EDS and the OEQ (Hausenblas and Downs, 2002; Pasman and Thompson, 1988). Next, themes were generated related to the various areas of life which could be impacted by this behaviour: social, work, health and injury. These themes were generated using prior case study research (Griffiths, 1997; Lyons and Cromey, 1989) and from research on overtraining syndrome (Kreher and Schwartz, 2012). Potential negative consequences within each of these domains were listed by the research team and verified with personal trainers who stated that they had personal or professional experience with problematic exercise (see Table 2). Finally, these items were pulled together and participants were asked to rate on a 5-point scale, how likely they would be to stop exercising if they experienced each of the consequences, ranging from Very Unlikely to Very Likely. The presentation of the questions to the participants was randomised.

**Table 2.** Factor analysis of exercise persistence scores, and internal reliability and descriptive statistics of the subfactors.

Domain	Consequence	Factor 1 (15.30% of variance)	Factor 2 (15.24% of variance)	Factor 3 (13.15% of variance)
**Work**	**You had lapses in concentration at work or place of education due to tiredness from your exercise**	**0.76**		
**Work**	**The quality of your work started to suffer because of your exercise regime**	**0.74**		
**Work**	**You were consistently late or missed deadlines due to the amount of exercise you were doing**	**0.70**		
**Work**	**Your manager suggested your performance was suffering**	**0.50**		
Social	Your exercise meant you would miss out on social activities	0.38	0.31	
Health	You were constantly tired and had low energy	0.35		
**Social**	**Your friends and family asked you to**		**0.65**	
**Social**	**Your partner raised concerns about the amount you were exercising**		**0.65**	
**Health**	**You had medical advice from a doctor to reduce your exercise regime**		**0.53**	
**Health**	**You experienced a change in your menstrual cycle (either irregularity or cessation) or a loss of libido**		**0.52**	
**Health**	**You experienced extreme changes in your resting heart rate and/or resting blood pressure**		**0.51**	
**Social**	**You lost contact with someone who used to be important to you because of your exercise regime**		**0.44**	
Health	You got repeated bouts of cold/flu like symptoms		0.32	
**Injuries**	**You experienced intense physical pain when exercising**			**0.82**
**Injuries**	**You experienced extreme pain which continues between exercise sessions**			**0.67**
**Injuries**	**You had recurrent injuries such as muscle sprains or stress fractures**			**0.66**
**Injuries**	**You were advised to stop exercising due to your injuries**			**0.48**
**Cronbach’s alpha**		0.79	0.77	0.80
**Possible scale range**		4–20	6–30	4–20
**Mid-point of scale**		12	18	12
**Mean sample score**		11.01	16.65	7.48
**Standard deviation of sample**		3.42	4.76	3.09
**% of sample scoring above mid-point**		33.81	33.09	7.91

Items in bold were retained after the factor analysis.

### Measures of biases

#### Learning bias (reward vs punishment sensitivity)

This was assessed using the BIS/BAS scales (Carver and White, 1994) which consist of 20 self-report questions measured on a 4-point Likert scale, covering sensitivity to punishment (e.g. I worry about mistakes) and sensitivity to reward (e.g. when I get something I want, I feel excited and energised). In this study, both scales had good internal reliability (BIS α = 0.82, BAS α = 0.81).

#### Cognitive bias (delay discounting)

This was assessed using the Zimbardo Time Perspective Inventory (ZTPI; Zimbardo and Boyd, 1999); a 56-item questionnaire that assesses participants’ time orientations across past-negative, present-hedonistic, future, past-positive and present fatalistic categories. For this survey, only the present hedonistic and future subscales were used. These scales had good internal reliability (present hedonistic scale α = 0.85, future scale α = 0.78).

#### Emotional bias (sensation seeking)

This was assessed using the Brief Sensation Seeking Scale (BSSS); a short measure of sensation seeking, consisting of eight questions measured on a 5-point Likert scale (Hoyle et al., 2002). This study found adequate internal reliability, with a Cronbach’s α of 0.74.

## Data screening

The survey data were exported to Jamovi 2.3.21 for statistical analysis. Metrics of exercise amount (moderate, vigorous and total hours), problematic exercise (medical model, subjective and objective problematic exercise) and biases (learning, cognitive and emotional) were assessed for normality. The biases, medical model and objective problematic exercise scales were found to be normal using *z*-scores of ±3.29. Even with the most extreme outliers removed (*z*-scores of greater than 3), both vigorous and moderate activity hours, and therefore total weekly exercise, were found to be highly skewed. Subjective problematic exercise was also positively skewed.

## Data analysis

First, to develop the new measure of objective problematic exercise the internal reliability of the full scale was measured using Cronbach’s alpha. Following this, the factor structure of the scale was assessed using exploratory factor analysis with oblique/nonorthogonal rotation. The internal reliability of the three subscales were assessed using Cronbach’s alpha, followed by subscale descriptives. Next, the relationship between the different measures of problematic exercise (medical model, subjective and objective) and each of the key biases, were assessed using a correlation matrix.

Subsequently, to identify whether any of the biases predicted different types of problematic exercise, three multiple linear regression models were conducted, one for each type of problematic exercise (medical model, subjective and objective). These regression models included age, total weekly exercise, gender and the measures for each of the biases (learning, cognitive and emotional).

## Results

### Validation and factor analysis of the exercise persistence scale

The Cronbach’s alpha for the full scale was 0.88, suggesting good internal reliability. Three factors were identified in the exploratory factor analysis, the first contained predominantly work-related consequences, the second was a mixture of health and social consequences and the third related to physical pain. These are shown in Table 2. Three of the consequences were dropped from the analysis as they did not load onto any of the factors greater than 0.4, leaving a total of 14 possible consequences of problematic exercise. The internal reliability and descriptive statistics of the factors highlighted through exploratory factor analysis are also presented in Table 2.

The Cronbach’s alpha results in Table 2 suggest that the three sub-factors have good internal reliability. This full 14-item scale and the three sub-factors are described in the following section as a measure of objective problematic exercise.

### Correlations between different measures of problematic exercise

The correlation matrix (Table 3) showed that the medical model and subjective and objective measures of problematic exercise were significantly positively correlated. The strongest correlation was found between the medical model and subjective measure (*r* = 0.48, *p* < 0.001), followed by the medical model and the objective measure (*r* = 0.27, *p* = 0.001) and finally the subjective and the objective measure (*r* = 0.17, *p* = 0.049). Weekly vigorous exercise amounts positively correlated with problematic exercise using the medical model (*r* = 0.20, *p* = 0.017) and using the objective problematic measure (*r* = 0.24, *p* = 0.004). Moderate and total weekly exercise amounts only correlated with problematic exercise when using the objective measure (*r* = 0.21, *p* = 0.014 and *r* = 0.31, *p* < 0.001 respectively). Subjective problematic exercise was not correlated with the amount of exercise.

**Table 3.** Correlation analysis of medical model, subjective and objective measures of problematic exercise and amount of exercise.

Variable	1	2	3	4	5	6	7	8	9	10	11
1. Medical model problematic exercise	-										
2. Subjective problematic exercise	0.48***	-									
3. Objective problematic exercise	0.27**	0.17*	-								
4. Vigorous exercise	0.20*	0.03	0.24**	-							
5. Moderate exercise	−0.00	0.05	0.21*	0.09	-						
6. Total exercise	0.14	0.05	0.31***	0.76***	0.72***	-					
7. Reward sensitivity	0.19*	−0.03	0.00	−0.00	0.12	0.08	-				
8. Punishment sensitivity	0.24**	0.16	−0.26**	−0.03	−0.26**	−0.20*	−0.07	-			
9. Future orientation	0.08	0.01	0.08	−0.03	−0.12	−0.10	0.09	0.30***	-		
10. Present hedonistic orientation	−0.05	−0.09	0.13	−0.02	0.14	0.08	0.55***	−0.34***	−0.42***	-	
11. Sensation seeking	0.01	−0.05	0.12	−0.03	0.14	0.07	0.50***	−0.27**	−0.23**	0.63***	
Mean scores	30.48	8.04	35.14	7.55	5.01	12.55	37.41	21.42	46.60	45.96	22.86
SD	8.66	3.88	9.06	7.78	7.27	11.11	5.23	4.03	7.32	8.26	5.59
Possible range	9–63	5–33	14–70	/	/	/	13–52	7–28	13–65	15–75	8–40
Recorded range	9–53	5–26	14–62	/	/	/	20–48	12–28	28–64	21–70	10–36

Items in bold were considered significantly correlated.

* *p* < 0.05. **p < 0.01. ***p < 0.005.

### Correlations between different types of problematic exercise and the three biases

Correlations between types of problematic exercise and biases are also shown in Table 3. Reward sensitivity positively correlated with problematic exercise only when measured using the medical model (*r* = 0.19, *p* = 0.026). Greater problematic exercise was correlated with greater punishment sensitivity when measured using the medical model (*r* = 0.24, *p* = 0.005), and lower punishment sensitivity when measured using the objective measure (*r* = −0.26, *p* = 0.002). Subjective problematic exercise did not significantly correlate with any bias in this study but came close to significance with higher punishment sensitivity (*r* = 0.16, *p* = 0.055).

### The role of biases in predicting different types of problematic exercise

Multiple linear regression analysis (Table 4) found similar but not identical results to the correlation analysis. Greater amounts of total exercise predicted greater problematic exercise as measured by medical model (*β* = 0.19, *p* = 0.028) and the objective measure (*β* = 0.27, *p* = 0.002), but not the subjective measure (*β* = 0.09, *p* = 0.33). Greater problematic exercise as measured by the medical model was predicted by greater punishment sensitivity (*β* = 0.29, *p* = 0.002) and greater reward sensitivity (*β* = 0.26, *p* = 0.031). Objective problematic exercise was not significantly predicted by punishment sensitivity (*β* = −0.18, *p* = 0.053), but the trend was for a negative relationship as seen in the correlation analysis. The overall multiple linear regression model for the subjective measure of problematic exercise, was not significant.

**Table 4.** Multiple Linear Regression predicting each of the conceptualisations of problematic exercise, using demographics and scores on each of the bias scales as predictive factors.

	Model 1 Medical model problematic exercise			Model 2 Subjective problematic exercise			Model 3 Objective problematic exercise		
	*R*^2^ = 0.16			*R*^2^ = 0.05, *p* > 0.05			*R*^2^ = 0.15		
Predictor Variable	*B*	SE *B*	β	*B*	SE *B*	β	*B*	SE *B*	β
Age	−0.09	0.07	−0.11	−0.02	0.03	−0.05	0.02	0.08	0.02
Total weekly exercise	0.15	0.07	0.19*	0.03	0.03	0.09	0.22	0.07	0.27**
Gender Female-Male	−1.93	1.55	−0.22	−1.03	0.74	−0.27	−0.34	1.64	−0.04
Reward sensitivity	0.42	0.19	0.26*	0.01	0.09	0.01	−0.21	0.20	−0.12
Punishment sensitivity	0.62	0.20	0.29**	0.21	0.10	0.22*	−0.41	0.21	−0.18
Future orientation	−0.08	0.12	−0.06	−0.02	0.06	−0.04	0.10	0.13	0.08
Present hedonistic orientation	−0.13	0.14	−0.12	−0.02	0.07	−0.05	0.11	0.15	0.10
Sensation seeking	−0.06	0.17	−0.04	−0.01	0.08	−0.01	0.12	0.18	0.07

Items in bold were considered significant predictors in the regression models.

* *p* < 0.05. **p < 0.01.

## Discussion

### Describing problematic exercise

Problematic exercise has been researched in numerous studies, predominantly under the guise of behavioural addiction. The nature of problematic exercise, however, is nuanced according to individual differences in interpretations of consequences and the beneficial nature of exercise. This study, therefore, used a novel combination of problematic exercise concepts: the medical model of problematic exercise; a subjective problematic relationship with exercise, whereby the individual perceives exercise as being problematic for them; and an objective view of problematic exercise, which focuses on persistent exercise despite negative consequences to various life domains.

Previous research has demonstrated the negative consequences of a problematic relationship with exercise in many domains of life, from social through to health. The exercise persistence scale was developed and validated using this initial sample, with a factor analysis suggesting three sub-factors. The first related to persistent exercise despite consequences to work life, and the second was more of a hybrid of consequences, with no defining theme but included consequences to social life, external advice, and some health consequences. For these first two subfactors, approximately 33% of the sample agreed that they would continue exercise despite consequences in these areas. The final subfactor from the factor analysis was persisting with exercise despite pain or injury. Approximately 8% of this sample responded positively to questions relating to this factor, indicating that they would continue to exercise despite extreme pain. Exploratory analysis on this sub-population indicated surprisingly these individuals who are willing to continue exercising despite extreme pain and injury are not more likely to think that exercise is problematic for them and are not more likely to have higher medical model scores based on withdrawal or interference. This population would therefore be interesting to explore further. Factor 3 of the Exercise Persistence Scale reflects single items used in previous measures of persistent exercise (Hausenblas and Downs, 2002; Pasman and Thompson, 1988; Taranis et al., 2011) that highlight continuance despite injury, but the use of the other two subscales provides a more in depth method of capturing this complex behaviour, such as the impact on work or studies as highlighted in prior case studies (Griffiths, 1997), the non-adherence to medical advice as covered in the OEQ (Pasman and Thompson, 1988) or the less obvious health impacts, such as interruptions to the menstrual cycle (Duckham et al., 2012). It is suggested that these questions be re-validated to assess whether clearer distinctions can be found using a larger sample. This new measure could be used on research to explore the causes and consequences of objective problematic exercise in terms of persistence in the face of consequences. It could also be used clinically to assess those at risk of physical damage or those who are less likely to adhere to the recommendation to rest in the face of injury.

This study found small positive correlations between the three types of problematic exercise, supporting the hypothesis. These findings suggest these are three inter-related but distinct concepts for measuring problematic exercise, moving beyond the medical model of addiction to include alternative conceptualisations of problematic exercise such as exercise persistence, which could be explored further. Some exercisers may score highly on traditional addiction-based criteria, such as withdrawal symptoms, but do not necessarily continue to exercise despite negative consequences. Conversely, an exerciser may persist with exercise through extreme pain and potentially cause negative consequences to those around them, but not view this behaviour as problematic. The subjective feeling that exercise is problematic is not necessarily related to the amount of exercise. The definition of problematic exercise appears to be a question of perspective.

The correlation between subjective and objective problematic exercise was weaker than expected. This could possibly be due to a lack of insight into the problem by the exerciser. A gambling awareness and insight scale was developed to assess impaired illness awareness in gambling, finding that awareness of negative consequences was positively correlated with gambling severity and negative emotional states (Kim et al., 2022). For gambling addiction, awareness of potential consequences is related to the severity of the problem. However, this may be more complex in problematic exercise. Prior research found a strong correlation between exercise addiction and passion (Szabo and Kovacsik, 2019). Highly passionate individuals who enjoy their chosen sports or exercise are highly likely to prioritise these above other life demands. For this subgroup, what may appear to be problematic exercise from an external viewpoint may instead be a passion that the individual is reluctant to change and will actively choose to prioritise, without experiencing symptoms of behavioural dependence, compulsion or addiction.

### The biases

This study also aimed to investigate the role of learning-, cognitive- and emotional addiction-related biases in these three types of problematic exercise. An addiction-based framework would suggest correlations with high sensitivity to reward, low sensitivity to punishment, greater influence of present hedonism than future orientation, and high sensation-seeking. However, the results of this survey were more complex.

For many, a key facet of addiction is the presence of a reward system deficiency. Substances of abuse have been found to disrupt an individual’s reward processing. Numerous studies have found that heightened sensitivity to reward corresponds with an increased risk of addiction, increased cue-elicited urges, and greater difficulties in adhering to treatment (Bechara, 2005; Kambouropoulos and Staiger, 2001; Mestre-Bach et al., 2016). Prior research found reward sensitivity predicts exercise addiction in young Australian adults (Lyvers et al., 2023). Correlation and multiple linear regression analysis in this study indicated that higher reward sensitivity was related to higher levels of problematic exercise when using the medical model. However, this study found no significant relationship between subjective and objective problematic exercise measures and reward sensitivity. This result highlights that although reward sensitivity does have a key influence on medical model-based exercise behaviour, there are other patterns of problematic exercise behaviour which are not based on reward sensitivity. This discrepancy in the reward response across problematic behaviours highlights the continued need for research highlighting the difference between addictive behaviours and compulsive behaviours (Cook et al., 2014).

It was anticipated that high sensitivity to reward would be found in conjunction with low sensitivity to punishment, in line with previous research (Lyvers et al., 2023). This was found to be the case when using the objective problematic exercise measure. However, correlation and multiple linear regression analysis indicated that high sensitivity to punishment was the strongest predictive factor for the medical model of problematic exercise and subjective problematic exercise. Suggesting that both high and low punishment sensitivity can lead to potentially problematic exercise behaviour, depending on the definition of problematic exercise. The relationship between high punishment sensitivity and subjective problematic exercise highlights the importance of perspective of the problem; when an individual is highly sensitive to negative consequences, they are more likely to perceive their own relationship with exercise as problematic, even if exercise amounts are equivalent to that of another person who has a lower punishment sensitivity.

Reinforcement sensitivity theory, which is the theoretical basis for the BIS/BAS scale as used in this study, was developed to conceptualise the neurological origins of anxiety (Carver and White, 1994). Therefore, using this scale may highlight anxiety-prone behaviour rather than specifically learning from punishment. This may have led to a confound in this study whereby participants with higher medical model or subjective measure scores, were driven by high levels of anxiety, rather than high punishment sensitivity. Whereas those with high scores on objective problematic exercise could be instead characterised by low levels of anxiety. Future studies could seek to isolate the role of punishment in learning in a more controlled setting as opposed to self-report measures or include an additional measure for anxiety to rule out any interference effects.

Delay discounting is considered by some as a key predictor of addiction-like behaviour (Bickel et al., 2014). It was expected that higher levels of problematic exercise would be associated with steeper discounting and a stronger focus on the present moment, rather than the future. However, the associations between time perspective and the measures of problematic exercise were not significant in the correlation or multiple linear regression analyses. It may be that problematic exercise differs from other addictions, due to the need for future planning and the upfront effort required to perform the exercise, meaning it is less characterised by the steep delay discounting seen in substance use disorders (Bickel et al., 2014). Alternatively, the measures used in this study may have identified problematic exercise that is more akin to disordered eating or compulsive disorders such as OCD, which are not associated with steeper discounting rates (Steinglass et al., 2017). This study also did not find evidence to support previous research linking time perspective with amount of exercise (Daugherty and Brase, 2010). This could potentially be due to this present study having a broader, older, community sample, rather than a student sample from the prior research. It could also be due to the measurement of the exercise, with the prior measure using frequency of exercise, whereas this present study used total amount of time exercising. Similarly, from prior research, it was expected that higher levels of sensation seeking would be linked to problematic exercise (Franques, 2003; Habelt et al., 2023), however, this was not supported with the data from this study. The results of multiple linear regression did not indicate any relationship between sensation seeking and any of the measures for problematic exercise, or with amounts of exercise. Some exploratory analysis indicated that the role of sensation seeking may be sport specific, with participants in this study who listed climbing as a frequent activity, having significantly higher levels of sensation seeking. It may well be that exercisers utilise their chosen physical activity to fulfil their preference for heightened or reduced sensations, but there is no overall trend that typifies problematic exercise.

The relationships between the three different definitions of problematic exercise, and the reward and punishment bias, indicate that there are multiple distinct ways of understanding problematic exercise behaviour beyond the medical model, and these are differentially related to individual responses to positive/rewarding and negative/punishing feedback. Problematic exercise behaviour that is characterised by more of a medical model of addiction is characterised by a heightened sensitivity to both types of feedback both reward and punishment. Exercise behaviour that is felt by the individual to be problematic regardless of how much exercise they do is instead characterised by heightened punishment sensitivity. Conversely, problematic exercise behaviour that is characterised by persisting despite negative consequences is unrelated to reward seeking and is associated with a desensitisation to punishment.

#### Limitations

This study design was a self-report survey; therefore, although an effort was made to differentiate three different forms of problematic exercise, these were all self-reported and subject to responder bias. For convenience, a set of questionnaires was chosen as an approximation for the biases under investigation; however, these could be better understood in a more controlled setting, such as a cognitive task directly assessing learning from reward and punishment could help remove any confounds with anxiety, which may have been present in the BIS/BAS scale. Differentiated analysis of primary and secondary problematic exercise (those without and with associated disordered eating) was not within the scope of this study but could add an additional layer of understanding for the development of the condition.

## Conclusion

This study aimed to broaden the understanding of problematic exercise using three conceptualisations: a measure based on the medical model of addiction, including withdrawal and interference with life, a subjective perspective of exercise being problematic, and an objective perspective of problematic exercise. The findings suggest these three concepts are related, but independent which describe different manifestations of problematic exercise. These types of problematic exercise were then compared with three biases found in the addiction literature: sensitivity to reward and punishment, delay discounting and sensation seeking, with the aim of understanding the key drivers behind the development and maintenance of these concepts of problematic exercise. Where problematic behaviour reflects a more medical model approach individuals demonstrate heightened sensitivity to both positive and negative feedback, which drives more vigorous exercise. Those who subjectively feel their exercise is problematic overly attend to negative feedback, even without excessive exercise. More objectively problematic exercise behaviour appears immune to both positive and negative feedback but is associated with high volumes of moderate and vigorous exercise. This study highlights the complexity of problematic exercise and the more individualised nature of how it can manifest in different people. Future research could utilise this broader conceptualisation of problematic exercise focusing on the medical, subjective and objective components as a means to both identify the causes and consequences of this behaviour and identify those most at risk of causing further harm through persistent behaviour.
